# Proteomic Characterization of Spinal Cord Myelin in the Mouse

**DOI:** 10.1080/17590914.2025.2595945

**Published:** 2025-12-10

**Authors:** Oliver Schmitt, Hannes Kaddatz, Stefan Mikkat, Markus Kipp, Antje Schümann, Sarah Joost

**Affiliations:** a Institute of Anatomy, University Medical Center Rostock, Rostock, Germany; b Institute for Systems Medicine, University of Applied Sciences and Medical University, Hamburg, Germany; c Core Facility Proteome Analysis, University Medical Center Rostock, Rostock, Germany

**Keywords:** CNS myelin, mouse, oligodendrocytes, proteome, spinal cord

## Abstract

The myelin proteome is a critical structural and functional component of the central nervous system (CNS), undergoing dynamic remodeling throughout life. Pathological changes, such as those in multiple sclerosis, disrupt myelin integrity and lead to severe neurological deficits. Establishing a reproducible baseline of the CNS myelin proteome is therefore essential for monitoring alterations in disease models. Here, we present a comprehensive proteomic dataset of purified spinal cord myelin from healthy mice. Myelin fractions were isolated by preparative sucrose density centrifugation, followed by gel-free peptide separation and mass spectrometric analysis. Label-free quantification based on at least two tryptic peptides identified 725 proteins across six spinal cord samples. Comparison with previous large-scale datasets confirmed the robustness of our workflow. In particular, our dataset showed a 71% overlap with the 809 proteins identified by Jahn et al. using a highly similar proteomic approach. Importantly, there was near-complete agreement for canonical myelin proteins, validating both the specificity and reproducibility of our method. Beyond this shared core, our dataset contributed additional proteins, including axon- and glia-associated candidates, expanding the baseline repertoire of the spinal cord myelin proteome. In summary, this study establishes and validates a reliable workflow for spinal cord myelin proteomics and provides a reproducible reference dataset. While not yet including diseased tissue, this baseline is directly applicable to experimental models of demyelination and remyelination, offering a critical foundation for detecting and interpreting disease-related proteomic alterations in multiple sclerosis research.

## Introduction

Accurate isolation and purification of myelin sheath proteins from the mouse central nervous system is essential for assessing alterations in this compartment in animal models of multiple sclerosis (MS). MS is a degenerative demyelinating disease that affects the brain and spinal cord (Thompson et al., 2018). The adult neurological diagnosis (Kurtzke, 2000; 2005), and knowledge on the pathogenesis is incomplete (Chan, 2020; Rahmanzadeh et al., 2018; Stys & Tsutsui, 2019), and no treatments arresting the disease (Baldassari et al., 2019; Baldassari & Fox, 2018; Saleem et al., 2019) are available.

Early studies focused on focal and inflammatory white matter lesions (Bendfeldt et al., 2010; Eden et al., 2019; Evangelou et al., 2000; Murray, 2009; Valcarcel et al., 2020) beside focal plaque formation (Absinta et al., 2015; Prins et al., 2015; Smith et al., 2020) as well as gray matter demyelination resulting in neurodegeneration and cognitive impairment (Fisher et al., 2008). Halting neurodegeneration and promoting neuroprotection are among the target outcomes of current therapeutic strategies (Baldassari & Fox, 2018; Maillart, 2018). Pathogenetic mechanisms are in the focus of several studies (Baecher-Allan et al., 2018; Belbasis et al., 2015; Donati, 2020; Garg & Smith, 2015; Haider, 2015; Kalincik, 2015; Lassmann & van Horssen, 2011; Lemus et al., 2018; Martin et al., 2016; Michel, 2018; Mimpen et al., 2020; Olsson et al., 2017; Rahmanzadeh et al., 2018; Soldan & Jacobson, 2001; Steiner et al., 2001; Stephenson et al., 2018; Taghizadeh et al., 2020). MS starts as an inflammatory disease, but is driven at later stages by neurodegeneration (Fambiatos et al., 2020; La Mantia et al., 2012; Lublin et al., 2016). In part MS contradicts the T- and B-cell driven inflammation resting in the progressive disease (Frischer et al., 2009; Machado-Santos et al., 2018; Romme Christensen et al., 2013) probably possible through specific ligand-receptor combinations of axon guidance molecules (ephrins, semaphorins, netrins) which also regulate inflammatory responses (Lee et al., 2019). In a mouse model of MS we demonstrated that diffuse injury to white and gray matter triggers the recruitment of peripheral immune cells and, hence, focal inflammatory lesions (Rüther et al., 2017; Scheld et al., 2016) suggesting that focal inflammatory lesions and diffuse degenerative processes mutually influence each other. Underlying molecular and cellular mechanisms are, however, poorly understood (Friese et al., 2014; Milo & Miller, 2014).

Since neuropathological changes in MS mainly comprise demyelination (Lassmann, 2014; Lubetzki & Stankoff, 2014) with subsequent axon degeneration (Correale et al., 2017; Criste et al., 2014; Ohno & Ikenaka, 2019; Pan & Chan, 2017), the aim of this study is to find out which proteins of the myelin proteome (Han et al., 2013; Raasakka & Kursula, 2020) can be identified and whether these proteins have also been identified in comparable studies (Ishii et al., 2009; Jahn et al., 2009; 2020; Monasterio-Schrader et al. 2012; Roth et al., 2006; Vanrobaeys et al., 2005). In a paper by Jahn and coworkers (Jahn et al., 2009), protein identifications are summarized using a variety of methods, some from previously published papers. In this study, fractions were separated by 2D-IEF-SDS PAGE, allowing gel-based myelin proteome maps to be produced. In the latter work, more specific separations via 16-BAC gels and CTAB-PAGE are used to achieve a relatively accurate separation of myelin proteins. By means of identification with nanoscale 1D ultra performance liquid chromatography (1D-UP-LC) separation coupled to detection with a quadrupole time-of-flight (QTOF) mass spectrometer it was possible to provide a list of 294 proteins (342 proteins are summarized in tabular form in Jahn et al., 2009, which were recorded in consideration of comparable publications) in the myelin fractions (Jahn et al., 2009).

A recent study of myelin components in the mouse brain (Jahn et al., 2020) combined various proteomic approaches to identify 1,155 proteins. A subset of 809 proteins was identified and quantified by a gel-free UDMS^E^ approach which is highly comparable to our analysis, since the same type of nUPLC system with identical reversed-phase columns coupled to a Synapt G2-S mass spectrometer was used for peptide analysis. As Jahn et al. (2020), we performed data-independent acquisition with ion mobility separation as an additional dimension of peptide separation and drift time-dependent collision energy settings (Distler et al., 2014). Moreover criteria for raw data processing, peptide identification and post-identification processing were comparable, although we used the Progenesis QI for proteomics software instead of the combination of PLGS and ISOQuant software used by Jahn et al. (2020).

Furthermore, comparable methods for myelin purification by sucrose density centrifugation and osmotic shocks (English et al., 2012; Erwig et al., 2019; Ishii et al., 2018; Larocca & Norton, 2006; Nair et al., 2011; Panfoli et al., 2014) were used in the work of Jahn et al. (2020) and applied in our study. However, unlike the study of Jahn et al. (2020) and other preceding proteomic analyses of CNS myelin from mouse (Ishii et al., 2009), we prepared myelin from the spinal cord instead of the brain. Furthermore, we studied samples of the entire spinal cord of the mouse rather than specific reactive cell populations of the spinal cord in EAE models (Turvey et al., 2014).

Another difference is related to the method used for solubilization of proteins from the myelin sheet and subsequent in-solution digestion. Established protocols (Erwig et al., 2019; Jahn et al., 2020; Siems et al., 2020) apply a lysis buffer containing several detergents including ASB-14 for protein solubilization followed by an adapted, automated protocol of the filter-aided sample preparation method (Wiśniewski et al., 2009). However, without automation this protocol is rather time-consuming. Here we used a method adapted from Masuda et al. (2008), in which proteins are solubilized by trypsin-compatible sodium deoxycholate (SDC) which is removed after digestion by phase transfer (Kumar et al., 2018; Pappesch et al., 2017).

Similar detergent-based solubilization and filter-aided sample preparation approaches were also applied in the recent study by Siems and colleagues (Siems et al., 2025), which provides a highly relevant large-scale analysis of the mouse brain myelin proteome. While both studies applied sucrose density centrifugation for myelin isolation, our approach differs in two major respects: (i) we focused on spinal cord rather than brain-derived myelin, and (ii) we used sodium deoxycholate (SDC)–based solubilization with phase transfer (Kumar et al., 2018; Masuda et al., 2008; Pappesch et al., 2017) instead of detergent-rich lysis buffers. Including the Siems dataset in our consensus analysis therefore allows us to directly contrast spinal cord– and brain-derived myelin proteomes and to identify both overlapping and region-specific myelin proteins.

The summary of identified proteins from different studies and methods of Jahn and collaborators (Jahn et al., 2009; 2020) was used for comparison in our work. In addition, however, we have included further studies in our comparison in order to be able to better assign differences or similarities to the respective studies (Roth et al., 2006; Vanrobaeys et al., 2005). Since the identification methods of some of these studies differed considerably, we performed a consensus analysis of each individual study to identify common proteins of the myelin proteome.

## Material and Methods

### Animals

For this study, 6 postnatal wild-type C57/BL6/J mice (143 d) were used. Animals were kept at 22 ± 2 °C under a 12 h light/dark cycle with free access to water and standard diet. All animal-related procedures were conducted in accordance with the local ethical guidelines and the German federal animal welfare law.

Mice were anaesthetized with an overdose of ketamine (750 mg kg^−1^ i.p.) and xylazine (50 mg kg^−1^ i.p.) and 20 cc of ice-cold phosphate-buffered saline transcardially perfused (PBS).

### Isolation of Myelin Proteins

We adapted the methods for myelin protein fractionation starting from established and cited protocols (Erwig et al., 2019; Larocca & Norton, 2006). Our procedure is presented in the following.

#### Dissection and Preparations

The spinal cord was dissected and divided into portions and then the samples were frozen and stored at −80 °C. The samples were thawed in 0.3 M sucrose. A maximum of 50 mg of tissue per 1 mL of 0.3 M sucrose (50 mg/1 mL sucrose buffer for 2.2 mL Seton 5011 open-top polyallomer centrifuge tubes) was used.

The solutions for the gradient centrifugations were always freshly prepared. Per 100 mg, 6 mL of 0.3 M and 0.83 M sucrose and 54 mL of Tris-HCl buffer were required for 3 steps of centrifugation (see below). All glassware and centrifuge tubes were precooled to 0–4 °C, as was the rotor of the centrifuge.

#### Homogenisation

We had placed the spinal cord samples in a cold Dounce tissue grinder containing 1 mL of 0.30 M sucrose solution. Homogenization of tissue was performed by using five strokes of the loose pestle and five to seven strokes of the tight pestle, on ice. Note: The solution must not exceed a concentration of 50 mg of wet tissue per 1 mL of solution.

#### Density Gradient Centrifugation

We had layered 1 mL of homogenate over 1 mL of 0.83 M sucrose solution in ultracentrifuge tubes, and separated by ultracentrifugation (Optima TLX Ultracentrifuge with a TLS 55 Rotor, Beckman) 30 min at 4 °C for 21,000 rpm (29314 g [RCF(avg)]/37785g [RCF(max)]). Thin-walled tubes were used and tubes were filled to within 2 to 3 mm from the top (∼2 mL more of 0.30 M sucrose solution should be added). Then the layers of crude myelin, which form at the interface of the two sucrose solutions, were collected with a pipette and transferred to the cold Dounce homogenizer. The rest of the gradient was discarded.

If the sample mass was greater than 50 mg, this step (2.2.3) was repeated once. In the case of a sample mass greater than 100 mg, the sample was divided into parts less than 50 mg. Each of these samples was processed through the steps of density gradient centrifugation (2.2.3) followed by the steps of 2.2.4 (see below). With the resuspension step in 2.2.4, the samples that had been handled individually up to this point were pooled again.

#### Osmotic Shock of Myelin Fraction

We resuspended the combined myelin layers in 1.0 mL of Tris·HCl buffer (pH 7.45) by homogenization using five strokes of the loose pestle and five to seven strokes of the tight pestle. The osmotic shock and differential centrifugation removed small microsomal membrane fragments and cytoplasmic contaminants. Then the myelin membranes were transferred to a graduated cylinder, and the suspensions were brought to a final volume of 2 mL with Tris-HCl buffer solution (pH 7.45). In the following we give the conversion for the rotor we used (TLS-55) from Beckman Coulter, since we did not use a fixed angle rotor. This has the advantage that the supernatants to be pipetted can be better delimited. We transferred the suspension to 2 mL ultracentrifuge tubes and centrifuged the suspension 15 min at 21,000 rpm and 4 °C (29,314 g [relative centrifugal field RCF(avg)]/37,785g [RCF(max)]). The supernatant was very carefully pipetted off and discarded. Then the pellet was resuspended in 1 mL Tris-HCl buffer solution using a 1 mL pipette. A further 1 mL of Tris-HCl buffer solution was added and resuspension was repeated. The suspension was transferred to ultracentrifuge tubes and centrifugated 13 min at 9,000 rpm (5,384 g [RCF(avg)]/6,940 g [RCF(max)]) at 4 °C.

Again the supernatant was very carefully pipetted off and discarded. Then the pellet was resuspended in Tris-HCl buffer solution using a 1,000 mL pipette. 1 mL of Tris-HCl buffer solution was added and resuspension was repeated. The suspension was transferred to ultracentrifuge tubes and ultracentrifuged 9 min at 9,000 rpm (5,384 g [RCF(avg)]/6,940 g [RCF(max)]) at 4 °C. The supernatant was very carefully pipetted off and discarded.

#### Purification of Myelin from the Crude Myelin Preparation

The crude myelin pellets were resuspended in 1 mL of 0.30 M sucrose solution. Then 1 mL of this suspension was layered over 1 mL of 0.83 M sucrose solution in ultracentrifuge tubes and ultracentrifuge 30 min at 21,000 rpm at 4 °C (29,314 g [RCF(avg)]/37,785 g [RCF(max)]). Thin-walled tubes were used and tubes were filled to within 2 to 3 mm from the top.

We harvested the purified myelin from the interface with a pipette and suspended the combined myelin layers in Tris-HCl buffer solution of the Dounce homogenizer. Then we discarded the rest of the gradient. This was followed by washing out the sucrose solution and subjection of the myelin preparation to further purification by hypoosmotic shock and differential centrifugation as described in 2.2.4 (Osmotic shock of myelin fraction). We performed a third discontinuous gradient (see 2.2.6) followed by a cycle of hypoosmotic shock and differential centrifugation as described in 2.2.4 (Osmotic shock of myelin fraction) resulted in a slight increase in myelin purity.

#### Final Purification of Myelin

After the second round of osmotic shock and centrifugation we resuspended the myelin pellet in 1.0 mL 0.83 M sucrose solution and layered 1 mL of this solution with 1.0 mL of 0.3 M sucrose solution in ultracentrifuge tubes. Ultracentrifugation was performed by 21,000 rpm for 30 min at 4 °C (29,314 g [RCF(avg)]/37,785 g [RCF(max)]). The interphase was pipetted off and the pellet was resuspended in 1 mL Tris-HCl buffer using a 1,000 µL pipette. Then we added another 1 mL Tris-Cl buffer and continued resuspending.

Now step 2.2.4 was repeated once. Finally the supernatant was pipetted off and the pellets were frozen at −80 °C.

### Measurement of Protein Concentration

For measuring protein concentrations, 3 x 25 μL of a sample were solved in lysis buffer (12% (wt/vol) CHAPS, 2 M thiourea, 5 mM DTT, 7 M urea) (1:10), protein standard for calibration curve (Thermo Scientific, 23208, Waltham, MA, USA), or albumin standard as a control (Thermo Scientific, 23210) were mixed with 750 μL Bradford protein assay reagent (Sigma B6916). After vortexing the protein solutions and 10 min incubation in the dark, the sample was measured in cuvettes in a UV spectrophotometer (Ultrospec 1100pro, Amersham Bioscience) without movement at room temperature, absorbance at 595 nm. All samples were measured in triplicate. Independent controls (Thermo Scientific, 23208) (pre-diluted protein assay BSA within a range of 0.25 mg/mL up to 1.0 mg/mL and albumin standard solution with concentrations of 0.4, 0.6, 0.8 mg/mL) were measured repeatedly.

### In-Solution Digestion of Proteins

Aliquots of the myelin preparations were supplemented with stock solutions of ammonium bicarbonate (ABC), sodium deoxycholate (SDC), dithiothreitol (DTT), and water to obtain a final volume of 50 µL extraction buffer (50 mM ABC, 3% SDC, 20 mM DTT). The protein samples were incubated at 95 °C for 5 min and subsequently sonicated for 10 min using a bath sonicator. Alkylation was performed with 30 mM iodoacetamide for 20 min at room temperature. Thereafter 50 mM ABC and sequencing grade trypsin (Promega) were added to obtain an enzyme/protein ratio of 1:50 in a final volume of 320 µL. Digestion was performed at 37 °C for about 16 h. SDC was removed from the digest solutions using the phase transfer surfactant method (Masuda et al., 2008) according to Pappesch et al. (2017) by adding 320 μl of ethylacetate and 6.5 µL of 25% trifluoroacetic acid, rigorous mixing for 3 min and subsequent centrifugation at 12,000 g for 10 min to obtain aqueous and organic phases. 200 µL of the aqueous phase was collected using a gel loading tip. Finally, the peptide solutions were desalted with OASIS HLB 1 cc Vac Cartridges (Waters, Manchester, UK), concentrated using a centrifugal evaporator and dissolved with 2% acetonitrile in 0.1% formic acid for mass spectrometric analysis. Peptide concentrations were measured using the Invitrogen Qubit protein assay kit (Thermo Fisher Scientific).

### Analysis by nanoLC-HDMS^E^

Liquid chromatography-mass spectrometry analyses were carried out using a nanoAcquity UPLC system (Waters) coupled to a Waters Synapt G2-S mass spectrometer via a NanoLockSpray ion source as described (Joost et al., 2019). Mobile phase A contained 0.1% formic acid in water and mobile phase B contained 0.1% formic acid in acetonitrile. Peptide samples (150 ng) were trapped and desalted using a precolumn (ACQUITY UPLC Symmetry C18, 5 µm, 180 µm x 20 mm, Waters) at a flow rate of 10 µL/min for 4 min with 99.9% A. Peptides were separated on an analytical column (ACQUITY UPLC HSS T3, 1.8 µm, 75 µm x 250 mm, Waters) at a flow rate of 300 µL/min using a gradient from 3% to 32% B over 120 min. The column temperature was maintained at 55 °C. The Synapt G2-S instrument was operated in data-independent mode with ion-mobility separation as an additional dimension of separation (referred to as HDMS^E^). By executing alternate scans at low and elevated collision energy (CE) of each 0.6 s, information on precursor and fragment ions, respectively, was acquired. In low-energy MS mode acquisitions were performed at constant CE of 4 eV whereas drift time-dependent CE settings (Distler et al., 2014) were applied in elevated-energy MS mode. Samples were measured in duplicate.

### NanoLC-HDMS^E^ Data Processing, Protein Identification, and Quantification

Progenesis QI for Proteomics version 4.1 (Nonlinear Dynamics, Newcastle upon Tyne, UK) was used for raw data processing, protein identification and label free quantification. Normalization and matching of features (accurate mass retention time tags) between runs was performed to compensate for between-run variation in the LC separation and to avoid missing values in quantification. Peak picking parameters included (i) sensitivity set to automatic, and (ii) a maximum ion charge of +4. Peptide and protein identifications were obtained by searching against a database containing 17,068 reviewed protein sequences from Mus musculus (UniProt release 2021_02). Two missing cleavage sites were allowed, oxidation of methionine residues was considered as a variable modification, and carbamidomethylation of cysteines as a fixed modification. The false discovery rate was set to 1%. Peptides were required to be identified by at least two fragment ions and proteins by at least one peptide and five fragment ions. Subsequently peptide ion data were filtered to retain only peptides that met the following criteria: (i) minimum ion score of 5.5, (ii) identified in at least two runs within the dataset, or in just one run if the ion score exceeds 7.0, (iii) mass error below 13 ppm, or below 20 ppm if the ion score exceeds 7.0, and (iv) at least 6 amino acid residues in length. Identifications based on charge state deconvolution were removed. Using the Progenesis software, some ambiguous peptide assignments were manually resolved and outlier peptides whose expression differed significantly from the other peptides of the same protein were removed. Only proteins identified by at least two peptides of which one is unique were included in the analysis. Proteins were quantified by the relative quantification Hi3 method (Silva et al., 2006).

### Immunoblot Analysis

The myelin-enriched spinal cord samples (2.1 mg/mL) were dissolved 1:1 in Laemmli buffer (1.05 mg/mL). For immunoblotting, extracts (15 μg per lane) were separated by SDS-Page using 4-20% polyacryamide gels (Bio-Rad, Munich, Germany) and transferred to PVDF membranes (0.2 mm, 7 × 8.7 cm, Bio-Rad, Munich, Germany) and equillibrated followed by methanol, rinsing in aqua dest. and then transfer buffer resp. Towbin buffer (25 mM Tris, 192 mM glycine, pH 8.3, with 20% methanol (vol/vol)). Blotting was performed for 1 h at 100 V. PVDF membranes were washed with TBS-T buffer (Tris-buffered saline with Tween20) (buf028, Bio-Rad) followed by staining with 1% Ponceau S solution and dye removal using TBS-T. The blots were blocked with 5% non-fat dry milk powder (Bio-Rad 1241) in TBS for 30–60 min and incubated with the individual primary antibodies. The following antibodies were used (dilutions in 5% non-fat dry milk powder TBS are given in the brackets) for over night incubation at 4 °C on a shaker: monoclonal anti-MBP (1:500, ab7349, Abcam, Cambridge, UK), monoclonal anti-MAG (1:500, ab89780, Abcam, Cambridge, UK), monoclonal anti-PLP (1:500, MCA839G, Bio-Rad). After washing in TBS (4 x 15 min), membranes were incubated with secondary goat anti-mouse HRP conjugated antibody (G21040, Invitrogen) for anti-MAG or anti-PLP, secondary goat anti-rat HRP conjugated antibody (ab97057, abcam) for anti-MBP (1:2000) and visualized by the Enhanced Chemiluminescence (ECL) (Lumixx plus 250) procedure as described by the manufacturer (Biostep GmbH, Burkhardtsdorf, Germany). As a molecular marker, the Precision plus Protein All Blue Standard (Bio-Rad Laboratories, Inc., Hercules, USA) was used. The analysis of the Western Blot protein signals was performed and recorded by using the Proxima 2850, CL and UV fluorescence/chemiluminescence system (Biostep GmbH, Burkhardtsdorf, Germany) including its analysis software (ProXima AQ-4, Ref. 1.28/CLIQS, version 1.1). Gel documentation was performed by using a Proxima 2850 system with fixed zoom (1.8), iris (0.95) and focus (15) between 10 s to 2 min.

### Bioinformatics

STRING, MINT, IntAct (EMBL-EBI), and Database of Interacting Proteins (DIP) were used for protein-protein interaction analysis (PPI). We used the Search Tool for the Retrieval of Interacting Genes/Proteins database (STRING v11) (Snel et al., 2000; Szklarczyk et al., 2019) to construct the PPI network of myelin proteins. Our lists of identified myelin proteins are the input for STRING. Then STRING can search for the protein neighbor interactors, the proteins that have direct interactions with the inputted proteins. Furthermore, STRING can generate the PPI network consisting of all these proteins and all the interactions between them.

## Results

First, we verified the presence of canonical myelin proteins in the spinal cord–derived myelin fractions. In all blots, PLP, MBP, and MAG were consistently detected in both spinal cord lysates and myelin fractions. The negative control (liver lysates) showed no signal. Equal amounts of total protein were loaded (15 μg per lane). The spinal cord myelin preparations (Spc1, Spc2) showed band patterns highly similar to spinal cord controls.

Proteomic analysis identified 725 proteins by at least two peptides (Supplement 1) compared to 809 proteins in Jahn et al., 2020, with an exact match of 571 proteins (71%). 253 proteins could be assigned to corresponding proteins from Jahn et al., 2009 (Supplement 2). 26 “known myelin proteins” from Jahn et al., 2009 were compared; with one exception (Mal), all were found in our samples (Table 1). 16 of these 25 proteins were detected in all samples. The following proteins (Cd9, Cldn11, Gpm6b, Omg, Pllp, Rab3c, Tspan2) were not present in all six analyzed samples but were identified in most of them. The 25 regular proteins identified in the spinal cord samples are compiled in Table 1.

**Table 1.** The known 25 myelin proteins identified here in the spinal cord and in Jahn et al. (2009). Myelin and lymphocyte protein (MAL) was not identified in our samples.

Protein name	Gene
2’3′-cyclic-nucleotide 3′-phosphodiesterase	Cnp
CD81 antigen	Cd81
CD9 antigen	Cd9
Cell adhesion molecule 3	Cadm3
Cell adhesion molecule 4	Cadm4
Claudin 11	Cldn11
Contactin 1	Cntn1
Ermin	Ermn
Ezrin	Ezr
Myelin basic protein	Mbp
Myelin protein P0	Mpz
Myelin proteolipid protein	Plp1
Myelin-associated glycoprotein	Mag
Myelin-associated oligodendrocyte basic protein	Mobp
Myelin-oligodendrocyte glycoprotein	Mog
NAD-dependent protein deacetylase sirtuin-2	Sirt2
Neural cell adhesion molecule 1	Ncam1
Neurofascin	Nfasc
Neuronal membrane glycoprotein M6-b	Gpm6b
Oligodendrocyte-myelin glycoprotein	Omg
Opalin TMP10	Opalin
Plasmolipin	Pllp
Ras-related protein Rab-3A	Rab3a
Ras-related protein Rab-3C	Rab3c
Tetraspanin-2	Tspan2

We identified 227 proteins in our samples that were also reported by Jahn et al. (2009) as “newly identified myelin-associated proteins” (Supplement Figure 1; Supplement 2). Comparison of our 725 proteins (Supplement 1) with the 809 proteins of Jahn et al. (2020) showed 571 matches (70.6%) (Supplement 3). In addition, 196 proteins were unique to our dataset, not present in previously published CNS myelin proteomes (Ishii et al., 2009; Jahn et al., 2009; 2020; Roth et al., 2006; Vanrobaeys et al., 2005) (Supplement 4).

**Figure 1.**
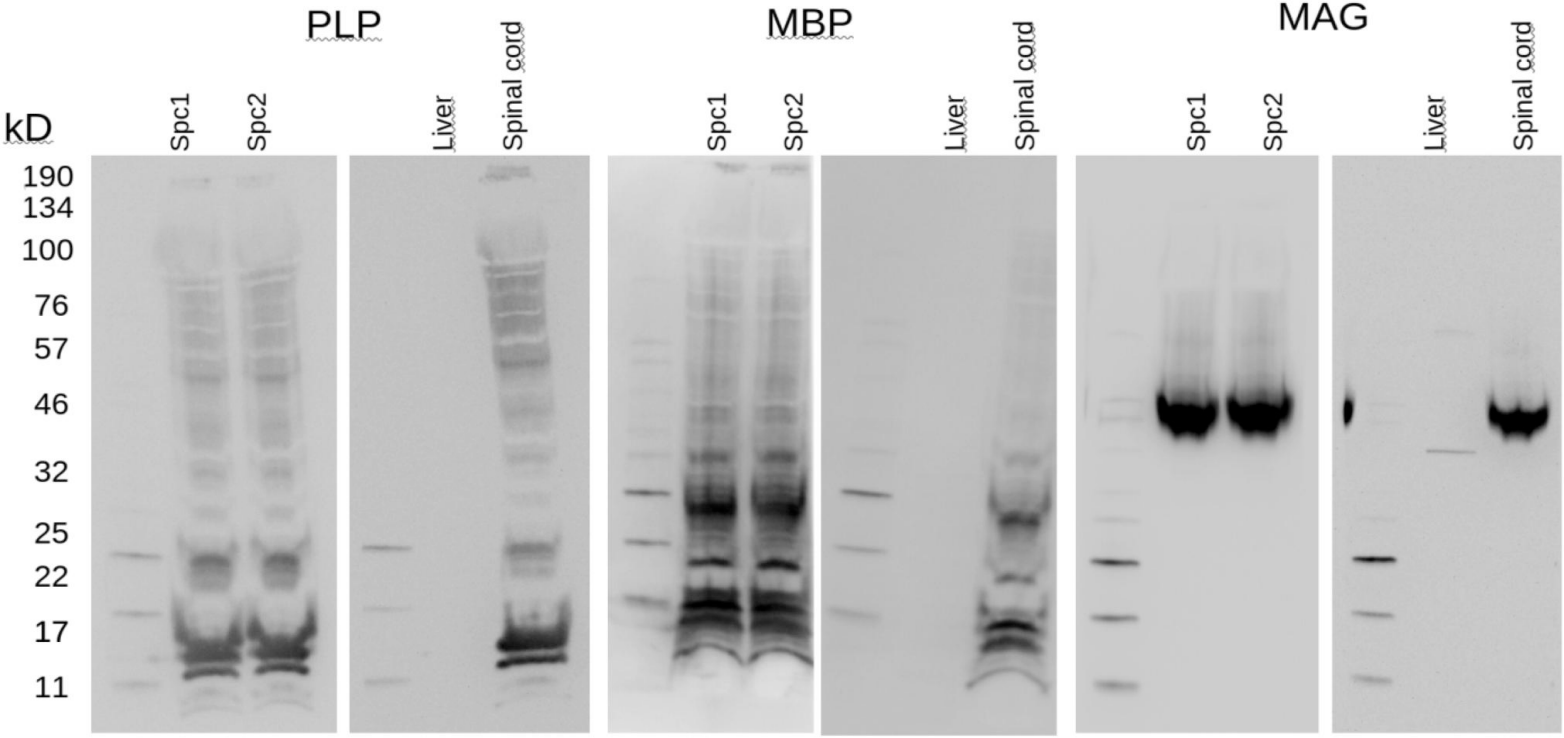
Western blots of PLP, MBP, and MAG. Each panel shows two pairs of blots. The left pair contains two spinal cord lysates obtained after isolation (Spc1, Spc2), run together to demonstrate reproducibility across independent preparations. The right pairs show a negative control (liver lysate) and a positive control (spinal cord lysate). A molecular weight marker was loaded in the first lane of each blot. Signals for the canonical myelin proteins are absent in liver lysates, confirming antibody specificity, and consistently present in both spinal cord lysates and enriched fractions, confirming preservation of myelin proteins.

Among the 196 novel proteins identified, 41 were functionally associated with terms linked to the axon and myelin sheath. From this subset, five proteins (Sncg, Aak1, Tbc1d24, Prrt2, and Ppp2ca) were consistently overrepresented across multiple axon-related Gene Ontology terms. STRING-based protein–protein interaction (PPI) analysis revealed that these five proteins are interconnected, forming a distinct cluster.

**Figure 2.**
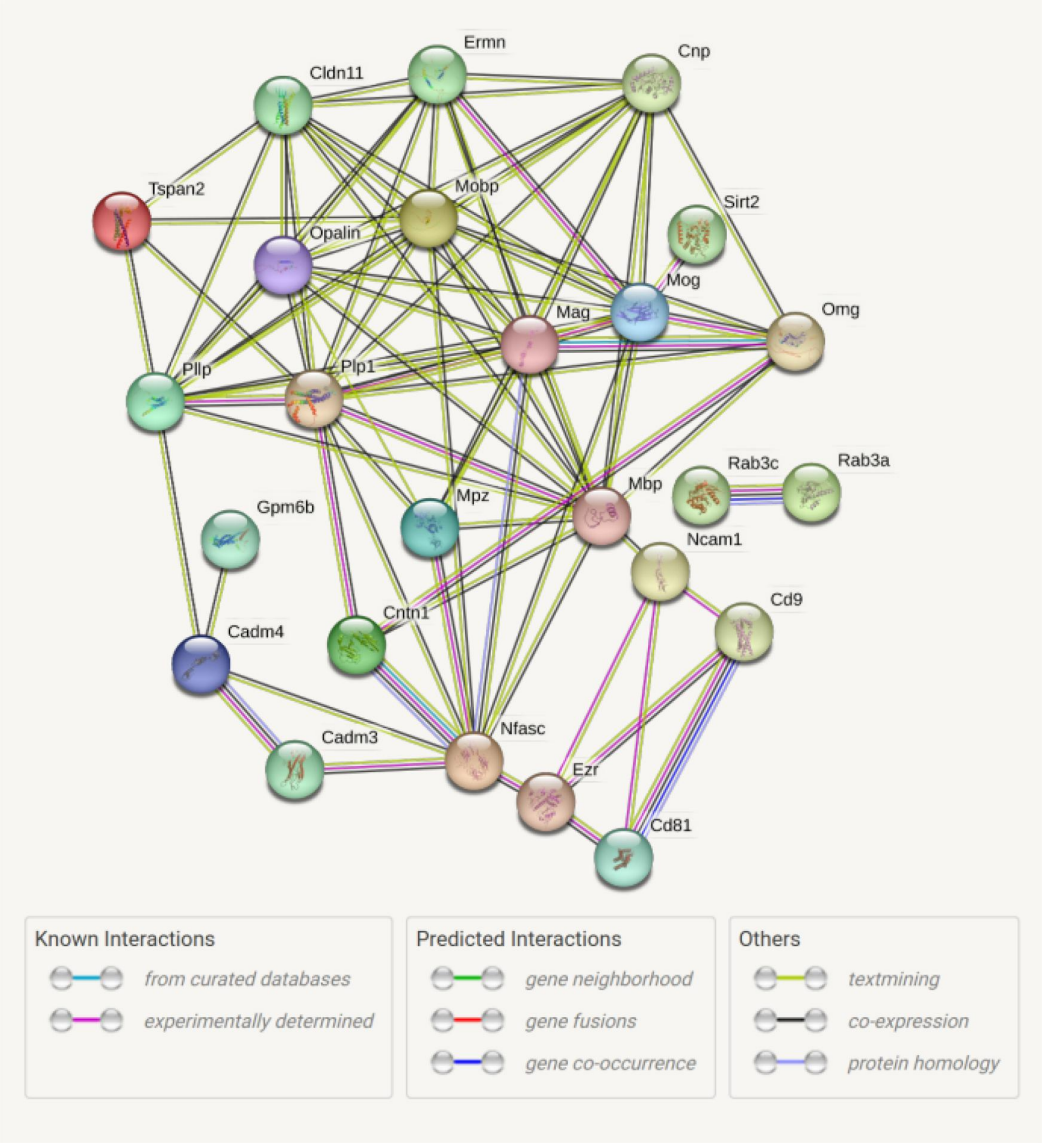
Interaction network of 25 regular myelin proteins identified in spinal cord myelin preparations. The network illustrates known and predicted protein–protein interactions; edges indicate functional associations, not necessarily direct physical binding. All proteins shown are well-established CNS myelin proteins with the exception of MPZ (Myelin Protein Zero), which is a PNS-specific protein likely included due to minor contamination from spinal roots during dissection. MPZ is retained here for transparency but should be interpreted as a peripheral myelin contaminant rather than a CNS myelin constituent.

The interaction of the 25 identified regular myelin proteins is shown in Figure 2. Two proteins were isolated, the two Ras proteins formed a 2-node subgraph, and the remaining 23 proteins were interconnected, forming a PPI network comprising 85 interactions. This network showed a mean cluster coefficient of 0.669, and functional overrepresentation analysis revealed associations with biological processes such as paranodal junction assembly, central nervous system myelination, axon ensheathment, and cellular response to low-density lipoprotein particles, as well as developmental processes including oligodendrocyte differentiation, regulation of myelination, axonogenesis, and neurogenesis (Herbert & Monk, 2017; Hughes, 2021). Cellular component assignments included the internode region of axons, the adaxonal region of the myelin sheath, and the paranodal region.

**Figure 3.**
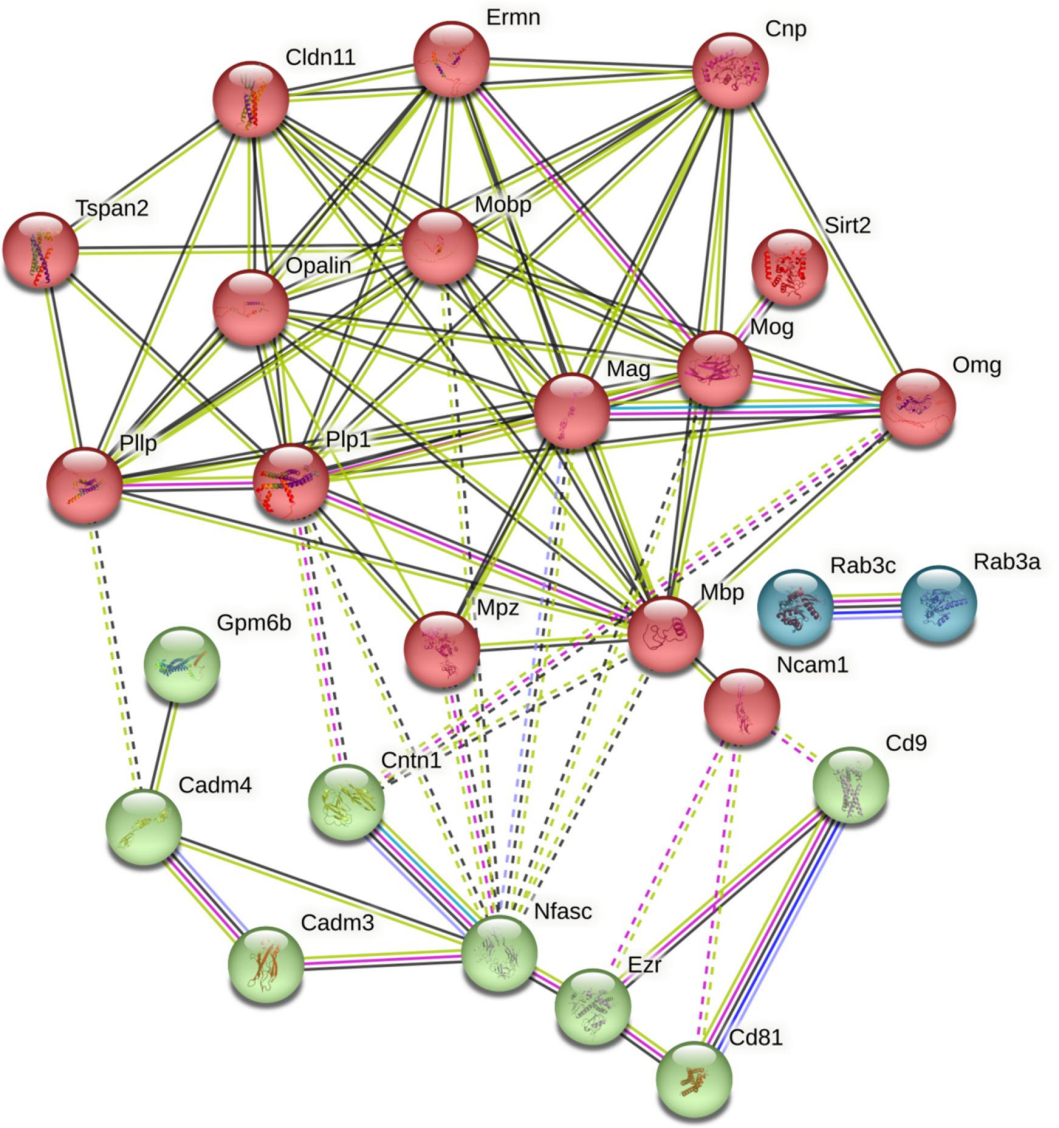
The 25 regular myelin proteins were assigned to three protein–protein interaction (PPI) groups by k-means cluster analysis applied to the STRING network. Unlike the standard STRING network (Figure 2), which shows all interactions together, k-means clustering highlights subgroups of proteins with denser internal connectivity and related functional roles. Cluster 1 (red) contains 15 proteins (axon ensheathment, myelin sheath), cluster 2 (green) contains 8 proteins (myelin assembly, synaptic membrane adhesion), and cluster 3 (blue) contains 2 proteins (anchored component of synaptic vesicle membrane, myosin V binding). Dotted lines indicate edges between clusters.

To refine the STRING network shown in Figure 2, we applied k-means clustering to the same set of 25 canonical proteins. This analysis partitioned the network into three subclusters (Figure 3). Within these clusters, the two proteins CD9 and CD81 of the cluster of differentiation antigens were detected. Ncam1 was also identified and showed protein–protein associations with contactin-1 (Cntn1) and neurofascin (Nfasc). Additional interactive relationships were observed between Nfasc and Cadm3, Cadm4, and Mpz. One cluster (green) comprised eight proteins (Cadm3, Cadm4, Cd81, Cd9, Cntn1, Mpz, Ncam1, Nfasc), with Nfasc located at the center. A second cluster (blue) contained Rab3a and Rab3c. The third and largest cluster (red) consisted of 15 regular myelin proteins, including Mbp, Mobp, Mag, Omg, Mog, Tspan2, Pllp, Sirt2, Ermn, Cntn1, and Gpm6b.

Among the identified proteins, myelin proteolipid protein (Plp/Plp1) was detected as a primary component of CNS myelin. In the PPI network, 2’,3′-cyclic-nucleotide 3′-phosphodiesterase (Cnp) occupied a central position with multiple associations to other proteins. Ezrin, a member of the ERM family, was detected in our dataset. Claudin-11 (Cldn11) was also identified.

We identified 227 proteins that were previously summarized as “newly identified myelin-associated proteins” by Jahn et al. (2009). Of these, 222 exhibited protein–protein interactions with each other (Supplement figure 1: STRING network of shared myelin-associated proteins from Jahn et al. (2009) and the current dataset). Many of the proteins clustered within cytoskeletal processes, including actin filament fragmentation, neurofilament assembly, and tubulin complex organization. Protein–protein interaction network analysis further revealed a significant overrepresentation of proteins annotated to the myelin sheath (GO:0043209), comprising 126 proteins from our dataset (FDR < 0.001).

Clustering of the 128 proteins annotated to the myelin sheath (GO:0043209), axon ensheathment (GO:0008366) and myelination (GO:0042552) revealed two major subclusters. The first contained 47 proteins functionally grouped under axon ensheathment, while the second comprised 55 proteins enriched for axon–glial interactions. Notably, this latter cluster included contactin-associated protein-like 2 (CNTNAP2) and periaxin (PRX), along with other candidates with established or putative roles at the axon–glial interface (Faivre-Sarrailh & Devaux, 2013). These clusters therefore highlight both structural and regulatory protein modules within the spinal cord myelin proteome (Supplementary Figure 3).

**Figure 4.**
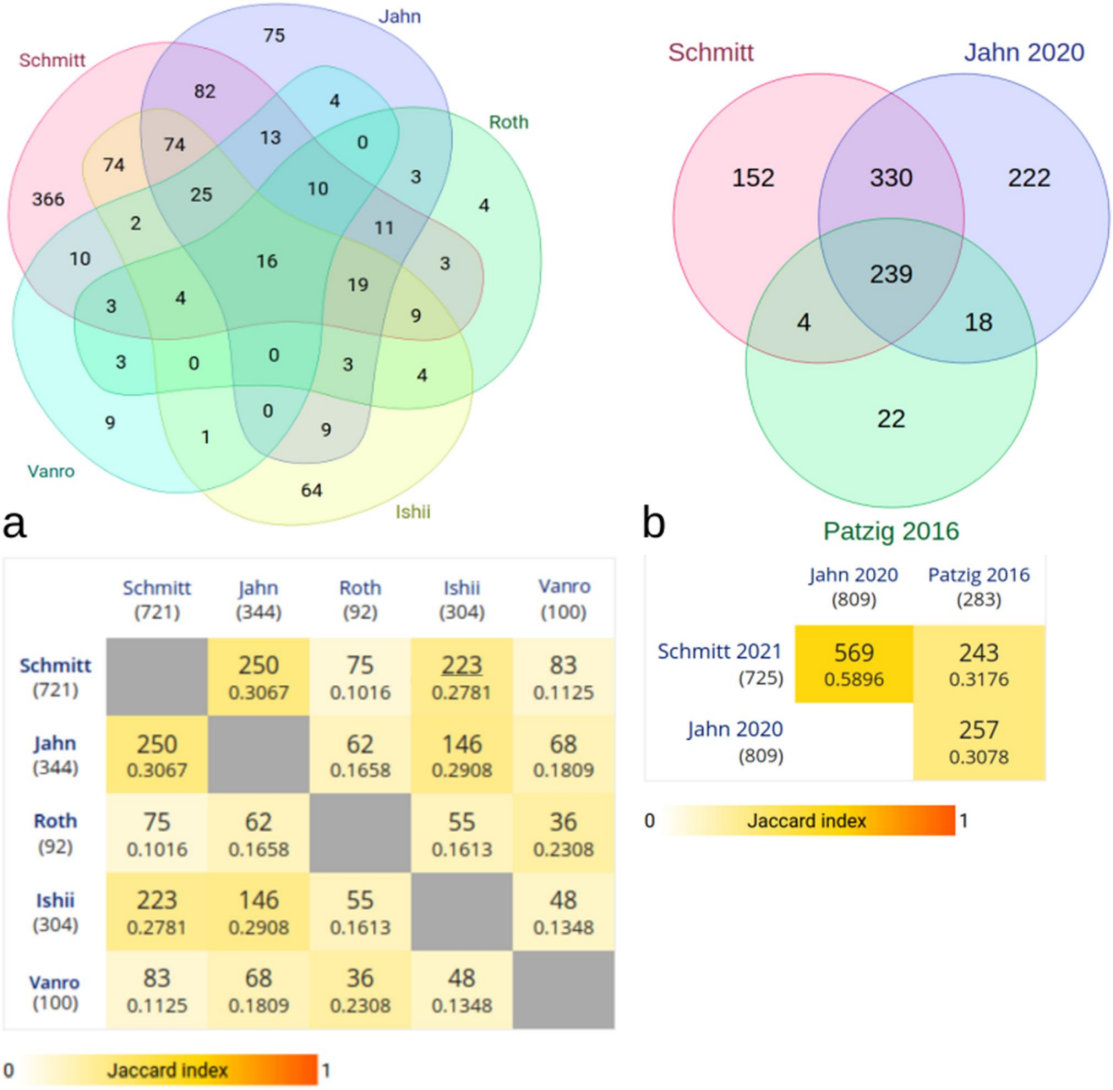
Comparison of myelin proteomes. a) Venn diagram of 5 myelin proteome studies (Ishii et al., 2009; Jahn et al., 2009; Roth et al., 2006; Vanrobaeys et al., 2005). Numbers represent shared protein identifications. The abbreviated label “Vanro” indicate author with a longer name. The adjacent matrix shows pairwise Jaccard similarity indices between datasets. Mitochondrial proteins, which copurify with myelin, were not excluded. b) Comparative overlap between our dataset and two recent studies: Jahn et al. (2020) and Patzig et al. (2016). Numbers represent protein matches; colors indicate Jaccard index.

To assess the novelty and coverage of our proteomic dataset, we compared our identifications with five published CNS myelin proteomes (Ishii et al., 2009; Jahn et al., 2009; Roth et al., 2006; Vanrobaeys et al., 2005). As shown in Figure 4a, our dataset shared 250 proteins with Jahn et al. (2009), followed by smaller overlaps with Ishii et al. and Vanrobaeys et al. The highest similarity was observed with Jahn et al. (2009), with a pairwise Jaccard index of 0.3067. Extending this comparison to more recent datasets (Jahn et al., 2020; Patzig et al., 2016) (Figure 4b), we identified 571 shared proteins with Jahn et al. (2020), corresponding to 70.6% overlap, and 243 shared proteins with Patzig et al. (2016). Across all datasets examined, 196 proteins were unique to our analysis (Supplement Figure 2: STRING network of proteins uniquely identified in the current spinal cord myelin dataset).

Across datasets, 16 proteins (ALDOA, ATP1A1, ATP1B1, CKB, CNP, DPYSL2, GSN, HSPA8, INA, MAG, MOG, NEFL, RAC1, SIRT2, THY1, YWHAZ) were consistently identified in all five studies. In addition, 74 matching identifications were found when comparing our proteins with those listed in Jahn et al. (2009) and Ishii et al. (2009).

A PANTHER overrepresentation test was performed to assign proteins to cellular components, molecular functions, and biological processes. The highest fold overrepresentation value (FE 29.36) was observed for mitochondrial proteins (Adriano et al., 2011; Bartolucci et al., 2015; Harris & Attwell, 2013; Morelli et al., 2011; Ravera et al., 2009; 2011; Roth et al., 2006; Taylor et al., 2004). Strong overrepresentation was detected for proteins associated with axons (FE 23.49), myelin sheaths (FE 22.74), internode regions (FE 22.02), and the juxtaparanodal region (FE 20.55) (Salzer, 2015; Wu et al., 2012). Proteins linked to pre- and postsynaptic compartments also showed FE values above 20, including postsynaptic endocytic zone cytoplasmic component, extrinsic component of the presynaptic endocytic zone membrane, anchored component of the synaptic vesicle membrane, postsynaptic intermediate filament cytoskeleton, and the presynaptic endocytic zone.

For biological processes, proteins were enriched in categories ranging from FE 10.21 to 4.57, including myelin assembly, CNS myelination, axon ensheathment, myelin maintenance, oligodendrocyte development, and oligodendrocyte differentiation. Regarding molecular function, proteins of the spinal proteome were assigned to the structural constituent of myelin sheaths (FE 11.74).

## Discussion

Proteome studies in MS research mainly focus on the cerebrospinal fluid proteome (Del Boccio et al., 2016; Hassan et al., 2016; Jankovska et al., 2020; Lee et al., 2016; Mosleth et al., 2021; Pavelek et al., 2016; Rosenling et al., 2012) for biomarker discovery (Linker et al., 2009; Teunissen et al., 2015) or on brain lesions of human MS (Bø et al., 2003; Maccarrone et al., 2017; Singh et al., 2019). In contrast, proteomic analyses in MS animal models (Palumbo & Pellegrini, 2017), such as experimental autoimmune encephalomyelitis (EAE) (Linker et al., 2009) and the neurotoxic cuprizone model (Wergeland et al., 2012), remain relatively rare (Hasan et al., 2019; Lereim et al., 2016; Lozinski et al., 2021; Oveland et al., 2021; Werner et al., 2010), and myelin proteomic analyses in these models have not yet been reported.

The purity of the myelin fractions was validated by complementary approaches. Western blotting confirmed the presence of canonical myelin proteins (PLP, MBP, and MAG) in both spinal cord lysates (positive control) and the myelin fractions (Jahn et al., 2020), while no signal was detected in liver tissue (negative control), thereby supporting antibody specificity and excluding nonspecific contamination. Although loading controls could not be included due to the limited availability of original material, the concordant detection of multiple canonical myelin proteins in independent assays strengthens confidence in the specificity of the fraction. Importantly, the proteomic analysis provided independent confirmation, revealing a strong representation of canonical myelin proteins relative to the tissue background. Together, the Western blots demonstrate specificity and preservation of myelin proteins, while the proteomics validates their prominent representation in the fraction analyzed (Figure 1).

Importantly, the proteomic analysis itself provided a second, independent validation: we detected 25 canonical myelin proteins described by Jahn et al. (2009), observed a 71% overlap with the large-scale myelin dataset of Jahn et al. (2020), and functional annotation analysis showed overrepresentation of proteins assigned to myelin sheaths, paranodal regions, and axon–glial junctions. Together, these results demonstrate that the sucrose gradient centrifugation procedure yielded a high-quality and reliable myelin fraction from the spinal cord.

The studies most similar to our myelin protein isolation method, which are also based on the myelin protein preparation of Larocca and Norton (Larocca & Norton, 2006), are those of Jahn and collaborators (Jahn et al., 2009; 2020). Therefore, we focused on the results of this research group and compared them with our identifications of proteins attributed to the myelin compartment.

We observed strong concordance with the datasets of Jahn and coworkers, including 25 canonical myelin proteins and an overall 71% overlap (Jahn et al., 2009; 2020). The remaining differences likely reflect tissue origin (spinal cord in our study versus brain in Jahn et al., 2020).

In addition to earlier myelin proteome studies, the recent large-scale analysis by Siems et al. (2025) provides an important reference point for brain-derived myelin. While both studies applied sucrose density centrifugation for myelin isolation, there are key methodological and biological differences that explain complementary outcomes. Siems and colleagues used detergent-rich buffers and filter-aided sample preparation, whereas our approach relied on sodium deoxycholate–based solubilization with phase transfer (Kumar et al., 2018; Masuda et al., 2008; Pappesch et al., 2017). This methodological difference may account for the detection of 196 proteins uniquely present in our dataset, including several hydrophobic and axon-associated proteins.

Beyond methodology, the most important distinction lies in the anatomical origin of the material. Siems et al. focused on brain myelin, while our work establishes the spinal cord–specific myelin proteome in mouse. This difference proved biologically informative: our dataset uniquely captured proteins involved in axon–myelin interactions and vesicle trafficking, including Sncg, Aak1, Tbc1d24, Prrt2, and Ppp2ca, which clustered together across multiple axon-related GO terms. Such proteins were absent in Siems et al., underscoring possible region-specific regulation of axon–glial communication in the spinal cord. In addition, we identified proteins generally associated with the PNS myelin proteome (periaxin, P2, nidogen-1) that were not reported by Siems. While some of these may reflect inclusion of spinal roots, they also align with the well-documented participation of Schwann cells in spinal cord remyelination (Duncan & Hoffman, 1997; Fex Svennigsen & Dahlin, 2013; Harrison, 1980, 1987; Itoyama et al., 1983, 1985; Kegler et al., 2015).

Direct comparison of the spinal cord–derived myelin proteome presented here with the brain-derived dataset of Siems et al. (2025) revealed a shared core of 446 proteins (61.8% of the Schmitt dataset; 41.6% of Siems; Jaccard index 0.331), underscoring the specificity of both fractions. Beyond this consensus, our dataset highlighted spinal specializations at the axon–glial interface and in excitability support, including proteins such as HCN2, CNTNAP1/2, CNTN2, ERMN, TSPAN2, CLDN11, MOG, and ENPP6. By contrast, Siems et al. expanded coverage of extracellular matrix and PNS-associated entries. These complementary signatures suggest that spinal cord and brain myelin differ in their proteomic emphasis, with the Schmitt dataset pointing toward spinal-focused candidates (HCN2, CNTNAP2, ENPP6, ERMN, TSPAN2) that warrant targeted validation. Together, the integration of both datasets advances the definition of a consensus pan-CNS myelin proteome and provides a framework for exploring region-specific contributions to demyelination and repair.

Taken together, these observations demonstrate that the spinal cord proteome provides complementary information to brain-derived datasets. While Siems et al. (2025) defined a comprehensive baseline for CNS myelin in the brain, our work extends this knowledge by (i) validating a reproducible spinal cord–specific workflow, (ii) highlighting region-specific and axon-related myelin proteins not captured in brain datasets, and (iii) providing a more inclusive proteome that also retains matrix and blood-associated proteins potentially relevant in MS models with blood–brain barrier disruption. We therefore view integrating both datasets as a step toward a consensus pan-CNS myelin proteome, a critical resource for future studies of demyelination, remyelination, and neurodegeneration.

In addition to these regional and methodological distinctions, we made a deliberate choice regarding data inclusiveness. We did not remove matrix and blood proteins from the data set because they may cross over into the neuropil of the spinal cord due to EAE-induced disruption of the blood-brain barrier, and thus may represent a reactive component. Such proteins may be relevant to the evaluation of experimental studies that alter the blood-brain barrier and blood-liquor barrier. We retained these proteins because they may reflect region-specific expression in the spinal cord, improved extraction/detection, or less abundant components of the myelin proteome; their inclusion provides a more comprehensive baseline for future studies of myelin remodeling, particularly in models of demyelination and remyelination.

The STRING analyses included in this study provided an overview of possible functional associations and biological clustering within the proteomic dataset. Such visualizations are helpful for generating hypotheses about pathways or molecular complexes that may be of interest in subsequent studies. However, the identity and purity of the myelin fraction were validated independently by Western blotting and by comparison with established myelin proteomes (Jahn et al., 2009; 2020). The STRING networks should therefore be viewed as a complementary, exploratory perspective rather than as evidence of validation (Smirnova et al., 2023, Smirnova et al., 2021).

We have also reviewed the protein myelin studies of Roth (Roth et al., 2006), Ishii (Ishii et al., 2009; 2018) and Vanrobaeys (Vanrobaeys et al., 2005). However, the study by Monasterio-Schrader and co-workers (Monasterio-Schrader et al. 2012) was not included because the proteins identified in human material predominate in this study.

Building on our proteomic findings, several identified proteins are functionally linked to neuronal and myelin biology, including CNTN2, AAK1, TBC1D24, PRRT2, and PPP2CA, which are involved in axonal conduction, vesicle trafficking, synaptic transmission, and axonal growth regulation (Cheng et al., 2015; Clark & Ohlmeyer, 2019; Falace et al., 2014; Eastwoodet al. 2010; Rossi et al., 2016; Taoufiq et al., 2020). These proteins cluster in biological processes related to myelin structure and function—such as paranodal junction assembly, CNS myelination, and axon ensheathment—and are supported by STRING analysis as components of interconnected regulatory networks (Nave & Werner, 2021; Simons & Nave, 2015). Among the detected proteins, periaxin, myelin P2, and nidogen-1—typically associated with the peripheral nervous system—were also present. Their occurrence may result from partial inclusion of spinal roots during dissection or reflect biological overlap at the CNS–PNS transition zone (Alfieri et al., 2012; Kister & Kister, 2023; Siems et al., 2020). Similar observations in previous proteomic studies support this interpretation (Duncan & Hoffman, 1997; Franklin & Ffrench-Constant, 2017; Harrison, 1980, 1987; Itoyama et al., 1983, 1985; Jessen & Mirsky, 2016; Kegler et al., 2015). Overall, these findings underscore the strong functional association among myelin-related proteins revealed by STRING analysis while acknowledging potential minor PNS contributions to the spinal cord proteome.3

Nevertheless, several lines of evidence indicate that the majority of our dataset represents bona fide CNS myelin: (i) Western blotting validated canonical CNS myelin proteins (PLP, MBP, MAG); (ii) our proteome shows 71% overlap with the large-scale CNS myelin proteome reported by Jahn et al. (2020); and (iii) functional analyses revealed strong clustering within established CNS myelin compartments. We therefore interpret the few PNS-associated proteins as reflecting either minor technical contamination or, potentially, genuine biological processes such as Schwann cell–mediated remyelination in the spinal cord (Duncan & Hoffman, 1997; Harrison, 1980, 1987; Itoyama et al., 1983, 1985). Future validation by immunohistochemistry or additional biochemical fractionation will be required to clarify their precise localization. Importantly, transparent acknowledgement of this issue ensures that our dataset can serve as a reliable reference baseline while also highlighting promising candidates for future study.

The greatest similarity, 71%, was observed between our myelin proteome and that reported by Jahn and coworkers (Jahn et al., 2009; 2020). A total of 223 identical proteins were shared with the datasets of Ishii et al. (Ishii et al., 2009; 2018), 83 with those of Vanrobaeys et al. (Vanrobaeys et al., 2005), and 75 with Roth et al. (Roth et al., 2006). These findings indicate that a basic core similarity of myelin proteomes can be established, even when sampling strategies, processing methods, and identification procedures differ. Indeed, multiple cross-comparisons yielded 16 proteins consistently identified as myelin components across all studies, despite methodological variation.

While such comparative proteomics helps to define conserved myelin signatures, further integration with transcriptomic resources may provide deeper insights. Since comprehensive transcriptome datasets are now available to elucidate the cellular basis of myelin dynamics (Thakurela et al., 2016), correlative proteome–transcriptome analyses represent a powerful next step to better understand the regulation of individual myelin proteins and to investigate their roles in MS disease models.

We will also consider the synaptic proteome (Laßek et al., 2015; Zhu et al., 2018) in the subsequent study to determine the consecutive functional effects as a consequence of demyelination. A possible use of the data would be the simulation (Schmitt et al., 2011; 2012; 2016; Schmitt & Eipert, 2012) of the connectional spread of demyelination (Balk et al., 2014; 2015; Bermel & Villoslada, 2014; Puthenparampil et al., 2017) and the resulting predictions of disease progression (Schmitt et al., 2017).

In this context, it is important to distinguish our dataset from remyelination proteomes established in pathological conditions (Paes de Faria et al., 2021). Our study provides a reproducible baseline of the spinal cord myelin proteome under physiological conditions, which is essential for such comparisons. To illustrate this baseline, we highlight several representative myelin proteins and their functional roles.

Ras-related protein Rab-3A is required for exocytosis and regulation of a late step in synaptic vesicle fusion (Leenders et al., 2001), while Rab-3C is associated with protein transport processes and vesicular traffic (Chang et al., 2023; Fischer von Mollard et al., 1994). These proteins emphasize the importance of vesicle-mediated trafficking in myelin protein dynamics.

Among the core and structural myelin proteins, myelin basic protein (Mbp) may exert selective effects on the developing brain long before myelination, possibly as a component of transcriptional complexes, and has also been implicated in T-cell and neural cell communication pathways (Haas et al., 1993; Landry et al., 1996; Lashgari et al., 1990; Vassall et al., 2015). Myelin-associated oligodendrocyte basic protein (MOBP) is thought to contribute to compaction and maintenance of the myelin sheath by binding negatively charged acidic phospholipids in the cytoplasmic membrane (Yamamoto et al., 1999). Myelin-associated glycoprotein (MAG) functions as an adhesion molecule that promotes connections between myelinating cells and neurons by binding RTN4R (Nogo-66 receptor 1), RTN4RL2, and neuronal sialic acid–containing gangliosides (Liu et al., 2002; Pronker et al., 2016). Although not required for the initiation of myelination, Mag is essential for maintaining proper axon–myelin interactions and preventing axonal degeneration. The myelin sheath also contains the smaller component myelin-oligodendrocyte glycoprotein (MOG) (Peschl et al., 2017) and the cell adhesion molecule oligodendrocyte-myelin glycoprotein (OMG), which contributes to interactive myelination processes in the CNS (Vourc’h and Andres 2004; von Büdingen et al., 2015). Tetraspanin-2 (Tspan2) appears to regulate oligodendrocyte signaling during terminal differentiation into myelin-forming glia and may also stabilize the mature sheath (de Monasterio-Schrader et al., 2013; Gobert et al., 2009; Yaseen et al., 2017). Ermin (Ermn) is required for cytoskeletal rearrangements during the late wrapping and compaction stages of myelinogenesis and for maintaining the stability of the adult myelin sheath (Brockschnieder et al., 2006). It is further implicated in the maturation of late-stage oligodendroglia, the creation of myelin/Ranvier nodes, and the flexibility of myelin-associated structures in the adult CNS (Ziaei et al., 2022).

The most abundant structural protein of CNS myelin is proteolipid protein (PLP, also termed PLP1 or lipophilin). This 276–280 amino acid hydrophobic protein with four transmembrane regions is highly conserved (Ruskamo et al., 2022; Simons & Trajkovic, 2006) and is essential for the formation and long-term maintenance of the multilamellar myelin structure. Studies of PLP knockout mice showed that animals develop initially without overt abnormalities and live beyond one year, indicating that PLP is not indispensable for myelin formation but rather contributes to stability of the myelin membrane (Griffiths et al., 1998). With age, however, progressive neurodegeneration manifests as ataxia and paralysis, reflecting axonal dysfunction (Rosenbluth et al., 2006). Evidence also suggests that Plp decreases in MS progression (Adiele & Adiele, 2019), with disturbances of axon–glia interactions and membrane trafficking during myelination (White & Krämer-Albers, 2014). Moreover, Plp has been proposed as a potential target of T-cell and B-cell responses in the EAE model (Glatigny & Bettelli, 2018).

Additional regulatory and signaling proteins were also detected. Plasmolipin (Pllp) appears to contribute to myelination and may influence ion transport, as its incorporation into lipid bilayers leads to the formation of voltage-dependent, K^+^-selective ion channels (Shulgin et al., 2021; Yaffe et al., 2015). Sirtuin-2 (Sirt2), a NAD-dependent protein deacetylase, modifies internal lysines on histone and alpha-tubulin and acts on multiple transcription factors, thereby linking epigenetic regulation and cytoskeletal dynamics (Jablonska et al., 2022; Ma et al., 2022; Zhu et al., 2012).

Finally, several adhesion and membrane-associated proteins further highlight the complexity of myelin organization. Contactin-1 (Cntn1) mediates cell surface interactions during nervous system development and has additional roles in oligodendrocytes (Çolakoğlu et al., 2014). Neuronal membrane glycoprotein M6-b (Gpm6b), a member of the proteolipid protein family, may be involved in SERT trafficking and regulation of serotonin uptake (Fjorback et al., 2009; Sanchez-Roige et al., 2022). Ezrin, a member of the ERM family (ezrin/radixin/moesin), links cytoskeletal elements to membranes and has been implicated in glial function (Derouiche & Geiger, 2019; Lavialle et al., 2011; Michie et al., 2019).

While the individual functions of these proteins highlight their diverse contributions to myelin structure and regulation, it is equally important to place them in the context of broader protein–protein interaction (PPI) networks. Beyond composition, we next examined potential functional relationships among the identified proteins. To explore potential biological relationships among the identified proteins, we performed a STRING analysis and visualized protein interaction networks (Smirnova et al., 2021) (Supplement Figure 3: STRING networks of spinal cord myelin proteins annotated to myelin sheath and axon ensheathment). This analysis was not intended as validation, but rather as an exploratory tool to illustrate how canonical myelin proteins and other components cluster into known functional groups. The networks thus provide a hypothesis-generating overview of potential interactions that can guide future targeted biochemical or immunohistochemical validation experiments. Collectively, these network-level observations complement the compositional comparisons above and help prioritize targets for targeted validation in spinal cord–focused MS models.

Many proteins were associated with cytoskeletal processes, including actin filament fragmentation, neurofilament assembly, and tubulin complex formation. Within these networks, we also identified proteins directly related to myelination that can be grouped into two functionally distinct clusters.

The first cluster comprises proteins involved in myelin synthesis and homeostasis. Choline transporter-like protein 1 (Slc44a1), a member of the CTL (choline transporter-like) family, participates in membrane formation and myelin development (Liu et al., 2025). The primary prion protein (Prnp), although not fully clarified in function, appears to play a role in neuronal development and synaptic plasticity (Cheon et al., 2024; Henzi et al., 2020), and it is likely required for maintaining neuronal myelin sheaths and promoting myelin homeostasis by acting as an agonist for the ADGRG6 receptor. Ectonucleotide pyrophosphatase/phosphodiesterase family member 6 (Enpp6), also known as choline-specific glycerophosphodiester phosphodiesterase, preferentially hydrolyzes lysosphingomyelin and converts lysophosphatidylcholine (LPC) into monoacylglycerol and phosphorylcholine (Morita et al., 2016). In addition, sphingomyelin—a sphingolipid highly enriched in mammalian cell membranes and particularly abundant in the myelin sheath—is directly linked to these metabolic pathways (Peter Slotte, 2013; Poitelon et al., 2020).

The second cluster includes proteins involved in axon–glial junctions and structural scaffolding. Contactin-associated protein-like 2 (CNTNAP2), together with CNTNAP1, is required for the radial and longitudinal organization of myelinated axons. This interaction supports the formation of functionally distinct domains that are essential for saltatory nerve conduction in myelinated fibers. The paranodal region of the axo-glial junction is defined by CNTNAP1, which mediates signaling between axons and myelinating glial cells in cooperation with contactin and may also contribute to delineating the juxtaparanodal region (Chang et al., 2023a). Periaxin, a scaffolding protein, further exemplifies this category: it acts in Schwann cells as part of a dystroglycan complex and in eye lens fiber cells as part of EZR- and AHNAK-containing complexes (Darmopil et al., 2008; Fieblinger et al., 2018; Gillespie et al., 1994). Periaxin is essential for the preservation of the peripheral myelin sheath, for proper nerve impulse transmission and sensory perception, and for MBP mRNA trafficking from perinuclear to paranodal regions (Wang et al., 2018).

By focusing on tissue-specific clusters and filtering out ubiquitous housekeeping pathways (e.g., glycolysis), the PPI analysis underscores the biological relevance of the captured proteome. These network insights not only reinforce the validity of our dataset as a reliable reference baseline but also highlight potential molecular targets for future studies of myelin pathology and repair mechanisms.

## Supplementary Material

Supplement_2_Comparison_clean.xlsx

Supplement_4_Presence_absence_matrix.xlsx

Supplement_1_Study_data.xlsx

Supplement figure 3.pdf

Supplement_3_Comparison_2.xlsx

Supplement figure 1.pdf

Supplement figure 2.pdf

## Data Availability

Not applicable.

## Data availability statement

The data mentioned in the paper are available in the text or as supplements.
