# Supplementary material for: Proteomic Characterization of Spinal Cord Myelin in the Mouse
Source: ASN Neuro. 2025 Dec 10;18(1):2595945. doi: 10.1080/17590914.2025.2595945 (PMC12795297; doi:10.1080/17590914.2025.2595945)
Supplement: Supplement figure 3.pdf [file TASN_A_2595945_SM8738.pdf]

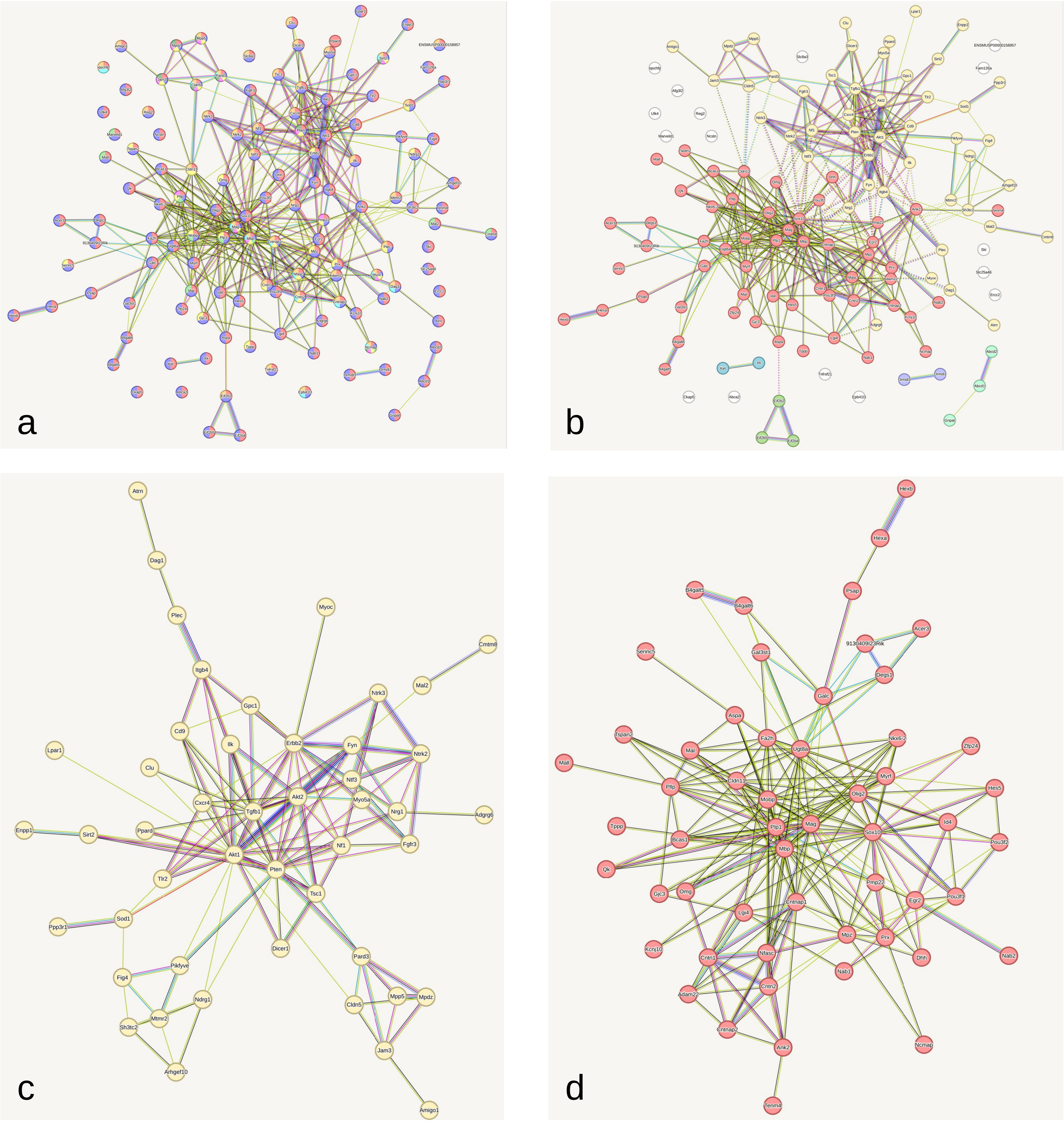

Supplement figure 3: Protein–protein interaction networks of spinal cord myelin proteins annotated to the myelin sheath and axon ensheathment. STRING network analysis was performed on 128 proteins annotated to myelin sheath (GO:0043209), axon ensheathment (GO:0008366), and myelination (GO:0042552). (a) Global interaction network of the 128 proteins associated with myelin and axon ensheathment/myelination. (b) The same network visualized with k-means clustering (k=6); node color indicates cluster assignment. (c) Subcluster 1, comprising 47 proteins functionally grouped under axon ensheathment. (d) Subcluster 2, comprising 55 proteins enriched for axon–glial interactions. Notably, the latter includes contactin-associated protein-like 2 (Cntnap2) and periaxin (Prx), together with additional candidates with established or putative roles at the axon–glial interface. These interaction modules highlight distinct structural and regulatory components within the spinal cord myelin proteome.

Axon ensheatment
Myelination
Myelin sheat
Compact myelin
Schmidt-Lanterman incisure
Main axon
Axon
