# Supplementary material for: Proteomic Characterization of Spinal Cord Myelin in the Mouse
Source: ASN Neuro. 2025 Dec 10;18(1):2595945. doi: 10.1080/17590914.2025.2595945 (PMC12795297; doi:10.1080/17590914.2025.2595945)
Supplement: Supplement figure 1.pdf [file TASN_A_2595945_SM8736.pdf]

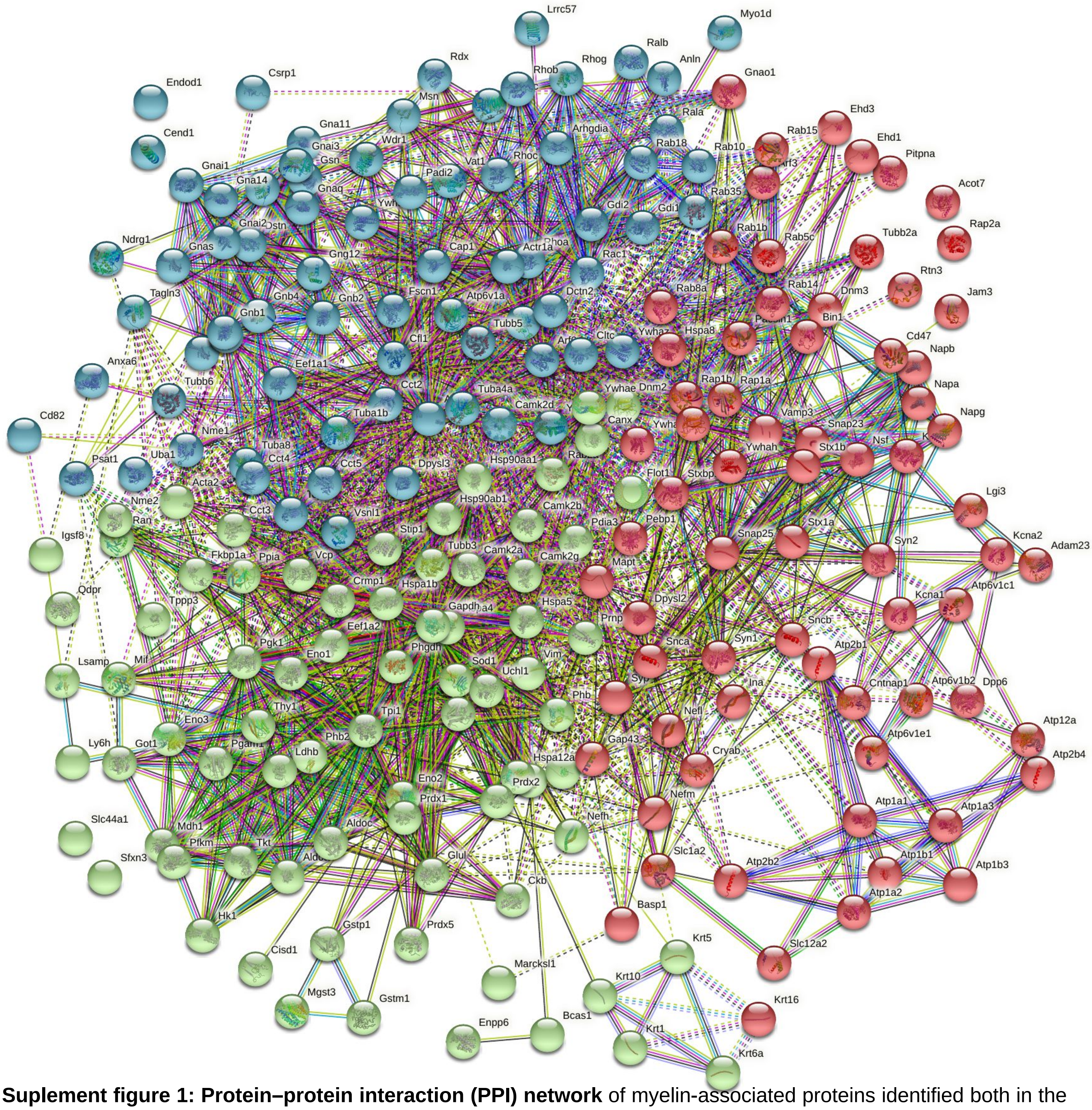

Suplement figure 1: Protein–protein interaction (PPI) network of myelin-associated proteins identified both in the Jahn et al. (2009) dataset and in our spinal cord samples. In total, 227 shared proteins were detected, of which 222 could be mapped into the STRING interaction network and are displayed here. The network was partitioned by k-means clustering into three groups of densely interconnected proteins (green, red, and blue). This STRING visualization is presented as an exploratory, hypothesis-generating tool to highlight potential functional associations among the shared proteins, and is not intended as experimental validation.
