# Supplementary material for: Proteomic Characterization of Spinal Cord Myelin in the Mouse
Source: ASN Neuro. 2025 Dec 10;18(1):2595945. doi: 10.1080/17590914.2025.2595945 (PMC12795297; doi:10.1080/17590914.2025.2595945)
Supplement: Supplement figure 2.pdf [file TASN_A_2595945_SM8735.pdf]

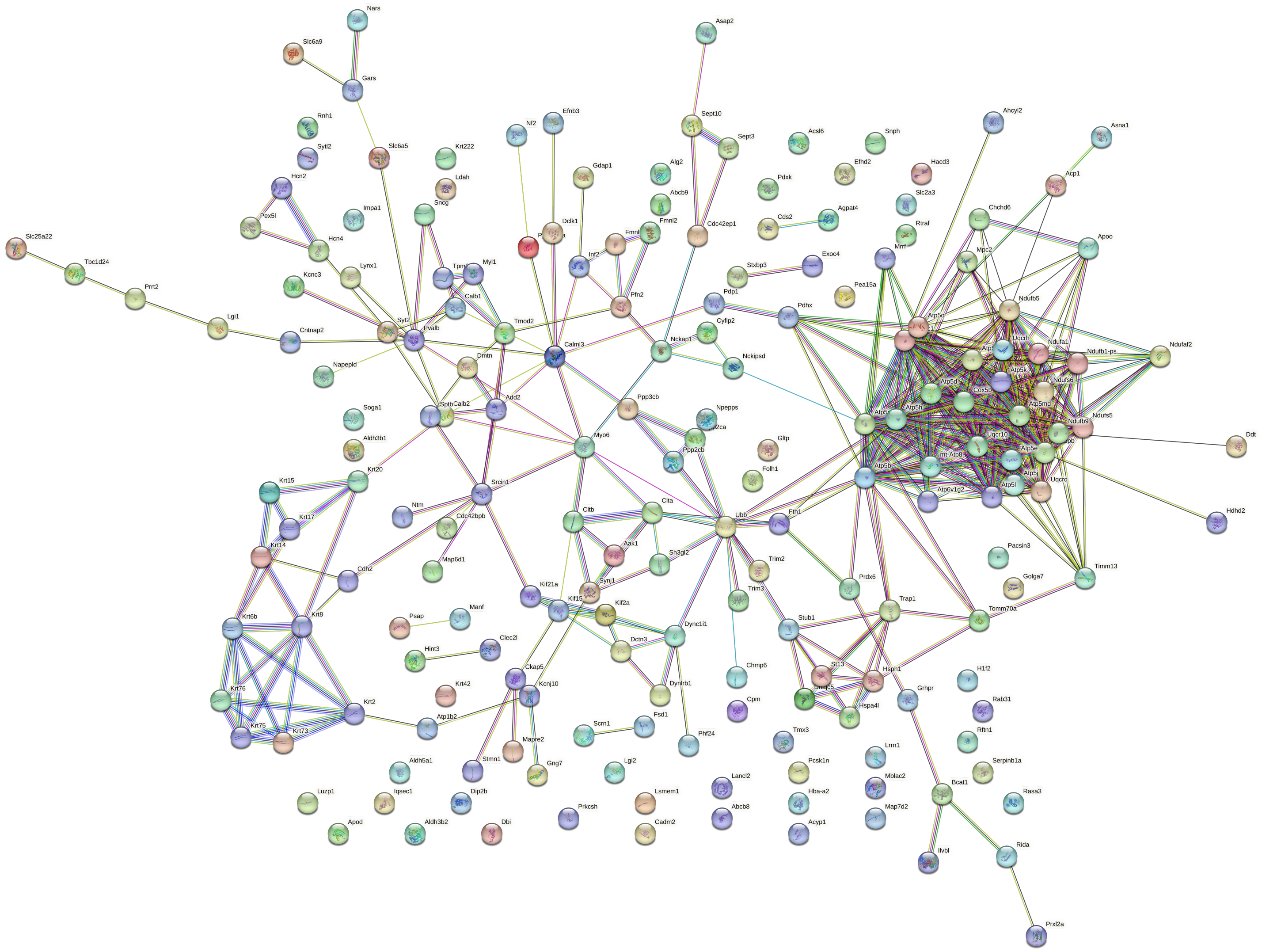

Suplement figure 2: This STRING network displays the protein–protein interactions among 196 proteins uniquely identified in our dataset, which were not detected in previous CNS myelin proteome studies (Jahn et al. 2009; Jahn et al. 2020; Ishii et al. 2009; Roth et al. 2006; Vanrobaeys et al. 2005). The network was generated using the STRING database with default confidence settings, and includes both experimentally validated and predicted functional associations (e.g., co-expression, gene fusion, and text mining). Each node represents a protein, and edges indicate known or predicted interac tions. A subset of 41 proteins (21%) is functionally enriched for myelin sheath and axon-related Gene Ontology (GO) terms, such as axon terminus, distal axon, and myelin maintenance. These proteins are distributed across the network and show variable connectivity, reflecting their diverse roles in axon–glial interactions, vesicle trafficking, cytoskeletal organization, and metabolic support. Densely connected clusters suggest potential functional modules, whereas peripheral nodes may represent novel or context-specific proteins with limited prior annotation. The network provides a foundation for identifying candidates involved in previously uncharacterized aspects of axonal insulation and support in the spinal cord myelin compartment.
